# A demonstrative study of a novel approach for spectral based source apportionment of ambient aerosols

**DOI:** 10.1038/s41598-025-04022-3

**Published:** 2025-06-04

**Authors:** Csilla Gombi, Abdul Rahman, Szabolcs Hodovány, István Magashegyi, Zoltán Bozóki, Gábor Szabó, Tibor Ajtai

**Affiliations:** 1 Department of Optics and Quantum Electronics, University of Szeged, 9. Dóm Square, Szeged, 6720 Hungary; 2 HUN-REN-SZTE Research Group for Photoacoustic Monitoring of Environmental Processes, Dóm ter 9, Szeged, 6720 Hungary

**Keywords:** Environmental impact, Environmental sciences, Health care, Optics and photonics, Physics

## Abstract

We present an alternative, spectral-based source apportionment model for calculating the mass concentration of wood burning and fossil fuel aerosols in the ambient. The model was applied to data collected from a rural area in Hungary during a 2-month continuous campaign in the winter period. The proposed model is based on the parallel measurement of the size distribution, absorption response of the ambient aerosols, and the thumb-of-rule relation between the elemental carbon (EC) and total carbon (TC) of fossil fuel and wood burning aerosols. We have experimentally demonstrated the wavelength dependency of the calculated Absorption Ängström Exponent (AAE) from the measured optical absorption coefficient (OAC) using the filter-accumulated transmission measurement method for the first time. The proposed model is compared with the traditional Aethalometer model applied to the same dataset. For comparison purposes, different but widely accepted approximations are used to determine source-specific AAE data and for the investigation of the effect of AAE on the output of the applied source apportionment models. The output of the applied models resulted in a close match with each other using site and source-specific AAE data.

## Introduction

The black carbon (BC) primarily consists of light-absorbing carbonaceous (LAC) particulate matter, which possesses significant optical absorption across the visible wavelength range. A significant portion of atmospheric BC concentrations originates from anthropogenic activities and can be characterized through its continuously varying spatial and temporal properties^1^. The BC plays a significant and unique role in the Earth’s climate system. Moreover, it is responsible for adverse public health effects and is recognized as one of the strongest indicators of the health effects of air pollution^2,3^. The BC absorbs solar radiation, accelerates ice melting, alters cloud formation, and contributes to a net positive radiative forcing on Earth’s climate^4,5^. The radiative forcing linked with carbonaceous particulate matter (CPM) remains a significant source of uncertainty in modeling Earth’s radiative budget^4,6^. The major contributors to LAC are fuel combustion, biomass burning, and biogenic emissions. The mixing state of BC is influenced by atmospheric chemistry through its atmospheric residence time and emission sources and combustion conditions^7,8^, which therefore, complicates the interpretation of the actually measured quantities, including light absorption by aerosols^9^. The characterization and quantification of aerosols originating from different sources are crucial for understanding the health and climate impacts of atmospheric BC^10^. Moreover, the chemical composition and concentration of LAC can vary significantly over short periods^11,12^, making it challenging for source apportionment models to accurately characterize emission trends using off-line data. Indeed, this underscores the significance of utilizing real-time measurement techniques for accurate source apportionment results. Many off-line source apportionment techniques employing chemical analysis of filter samples have been demonstrated in the literature^13–15^. However, these techniques suffer from significant methodological drawbacks, such as long analysis times and poor time resolution. Consequently, these limitations result in the loss of valuable information about real-time changes. The chemical analysis, especially positive matrix factorization and chemical mass balance methods combined with a spectral-based real-time source apportionment model, strengthens the validity of the proposed novel source apportionment model. We used the Aethalometer model, which is widely accepted as an absorption-based source apportionment method, and well-confirmed by the chemical balance method^16–18^. Moreover, the direct comparison of spectral-based and chemical analysis-based source apportionment, especially the chemical mass balance method, gives an uncertainty range of 10% to 40%, mainly because of different measurement techniques and conceptual hypotheses made for each model^17^. Among all the physicochemical properties of LAC, only the AAE quantified by the slope of absorption response in log–log scale serves as a real-time measurable physical quantity with source-specific and composition relevancies^19,20^. Based on that, the past decades have witnessed the demonstration of spectral-based methodologies for source apportionment results^19,21,22^. The AAE aids in the identification of typical combustion-like aerosol sources such as wood burning (wb) and fossil fuel (ff) combustion, where the latter one dominantly originates from traffic activities^16^. The aerosols originating from the wood burning emission exhibit higher OAC towards the shorter wavelength and become dominant in the low visible and especially in the UV wavelength domain compared to particles originating from traffic emissions^23^. Taking advantage of these distinct characteristics of aerosols, the first spectral-based source apportionment method was demonstrated, which is a so-called Aethalometer model in the literature^19^. Although the Aethalometer instrument is commonly used for measuring light absorption of the aerosols, the deduced OAC from the measured transmission of the filter accumulated aerosols suffers from many analytical and methodological artifacts^24,25^. In the Aethalometer model, the source-specific AAE values are not known a priori. Defining site- and source-specific AAEs typically requires complex, offline measurements involving multiple analytical techniques, which often suffer from low time resolution. However, recent advancements in analytical methodologies and data integration strategies have demonstrated the potential to derive time-resolved AAE values without relying solely on offline techniques^26,27^. To address this pressing issue, an improved methodology is imperative for providing reliable data for source apportionment results, demonstrating significant improvements in data quality and the ability to accurately identify emission sources.

In this study, we proposed a simplified source apportionment model based on real-time and parallel measurement of the size distribution and optical absorption data. The proposed model relies on the rule-of-thumb relation between TC and EC mass of the different sources. We also explored the wavelength dependency of the AAE deduced from the OAC measured by the Aethalometer. The source apportionment results derived from the proposed model and the original Aethalometer model are compared. Finally, we also revealed and quantified the correlation between the AAE and other source-specific markers of the carbonaceous particulates.

## Measurement site and methods

### Measurement site

The data for this study were obtained from the K-puszta measurement station, Hungary (46° 58′ N, 19° 35′ E and 125 m above sea level), which is 15 km northwest of the nearest town, Kecskemet (70,000 population) and 80 km southeast of Budapest (1.7 million population). The site represents the main plain part of the Pannonian basin^28^ and is surrounded by 62% coniferous trees, 28% deciduous trees, and 10% grassland^29,30^. The measurements were taken during a campaign in winter from Dec. 25, 2017, to Feb. 25, 2018. The K-puszta station is exposed to polluted masses having higher particulate matter^31^, as well as the cleaner and colder air from the Atlantic Ocean. Moreover, it is strongly affected by biomass smoke from domestic heating during winter^32^.

### Instrumentation and sampling

All the instruments were placed inside the temperature-controlled facility at the K-puszta measurement station. The Aethalometer (Magee Scientific, USA, type AE31) was used to measure the OAC of ambient aerosols at the defined wavelengths: 370, 470, 520, 590, 660, 880, and 950 nm. The working principle of the Aethalometer is described in detail elsewhere^33^. The characteristic size distributions of the ambient aerosol particles ranging from 6 to 800 nm were measured by a differential mobility particle sizer (DMPS)^34^. The DMPS mainly consisted of a Nafion semi-permeable membrane dryer, a radioactive bipolar charger, a differential mobility analyzer (DMA), and a butanol-based condensation particle counter (TSI CPC3772). DMA classifies aerosols through electrical mobility. The copper tube of 4 mm diameter was used as a sampling line without any special upper-size cut-off inlet. The dried aerosols were collected in 30 channels with 1 LPM sheath flow and 0.3 LPM sample flow. The time resolution of the measurements was approximately 10 min. All the measurements were performed according to the international technical standard^35^. The particles were collected and analyzed continuously during the whole campaign. The OC/EC analyzer (Sunset Laboratory, USA, 4G-Semicontinuous Field Analyzer) was employed for elemental carbon measurements. Before site implementation, the analyzer underwent calibration according to the manufacturer’s specifications. A comprehensive explanation of the OC/EC analyzer’s operational principles can be found elsewhere^36^.

### The Aethalometer and its alternative source apportionment models

#### The Aethalometer model

The operational principle of the Aethalometer is well described in many studies^37–39^. Briefly, the sampled airflow, including the aerosol, is directed through a filter tape that captures aerosol particles. Optical filter photometers then measure the transmission of the filter-accumulated aerosol^40^. The absorption coefficient is then deduced from the coefficient of attenuation. The expression of light intensity for the unexposed portion of the filter tape (I_o_) is related to the intensity from aerosols accumulated part by Beer’s law:

1 $$I(\lambda ) = I_{o} exp\left( { - \ell {*}OAC\left( \lambda \right)} \right),$$

where $\ell$ is optical path length (the distance the light travels through the aerosol-laden portion of the filter tape), i.e. $\ell = \Delta t \times F{/}A$, with Δt, F, and A are aerosol deposition time, volumetric flow rate, and area of the deposited aerosol filter, respectively^41,42^. However, the filter attenuation measurements suffer from two major artifacts^43,44^. One is the shadowing effect, which arises when the embedded aerosols are superimposed on each other. The other is the so-called multiple scattering effect, which appears due to the deposition of aerosols at different positions of the filter matrix, resulting in multiple scattering between the embedded particles. To minimize these measurement artifacts, different posterior data treatments are used^45,46^. However, recent research has demonstrated that the correction factor for multiple scattering effects varies with wavelength and composition^46,47^.

The Aethalometer model uses the deduced OAC of aerosols from measured transmittance data. Given that wood-burning aerosols have lower absorption at near-IR as compared to absorption of aerosols generated from fossil-fuel burning, and show increased absorption towards the shorter wavelengths, resulting in higher absorption at the UV wavelength domain. The ultimate operational wavelengths of the instrument are used to selectively identify the emission sources (370 nm and 950 nm). The Aethalometer model is based on the two following assumptions: (i) the dominant sources of the carbonaceous aerosol are the wood burning (wb) originating primarily from the heating activity and the fossil fuel (ff) aerosols typically emerging from traffic activities, (ii) the AAE is a source specific marker of the two sources. These assumptions can be expressed by the following equations:

2 $$OAC\left( {370{\text{ nm}}} \right) = OAC_{ff} \left( {370{\text{ nm}}} \right) + OAC_{wb} \left( {370{\text{ nm}}} \right)$$

3 $$OAC\left( {950{\text{ nm}}} \right) = OAC_{ff} \left( {950{\text{ nm}}} \right) + OAC_{wb} \left( {950{\text{ nm}}} \right)$$

4 $$\frac{{OAC_{ff} \left( {370 \;{\text{nm}}} \right)}}{{OAC_{ff} \left( {950 \;{\text{nm}}} \right)}} = \left( {\frac{370}{{950}}} \right)^{{ - AAE_{ff} }}$$

5 $$\frac{{OAC_{wb} \left( {370 \;{\text{nm}}} \right)}}{{ OAC_{wb} \left( {950\;{\text{ nm}}} \right)}} = \left( {\frac{370}{{950}}} \right)^{{ - AAE_{wb} }}$$

To define the source-specific absorption coefficients (OAC_ff_ and OAC_wb_) from Eqs. 2–5, the priory not known AAE_ff_ and AAE_wb_ should be specified by auxiliary measurements. In the originally proposed Aethalometer model, the AAE_ff_ is fixed at 1.1 value, while the AAE_wb_ was determined using thermoanalytically corrected radiocarbon measurement data made on the filter-sampled particles with a 24 h time resolution^19^. In some other studies, simply the EC and OC data deduced from the thermoanalytically measured results are used for the determination of AAE_wb_ using the same procedure^48,49^. Moreover, in some measurement campaigns, non-site-specific values from the literature^50,51^, or even default values of AAEs (AAE_ff_ = 1 and AAE_wb_ = 2) are applied^52,53^. However, those assumptions impose a significant limitation on the accuracy of the results^54^. Moreover, some recent studies have experimentally demonstrated that the fixed AAE value of fossil fuel aerosols also imposes strong limitations for measurement accuracy^55,56^. With known AAE_ff_ and AAE_wb_, the contribution of the wood burning and fossil fuel aerosols to the measured OACs at a specific wavelength can be determined directly from Eqs. 2–5. After that, the contribution of wood burning and fossil fuel aerosols to total carbonaceous mass (CM) can be expressed by the spectral properties of the emissions associated with different sources

6 $$CM = CM_{wb} + CM_{ff} = c_{wb} \cdot OAC_{wb} \left( {370\;{\text{nm}}} \right) + c_{ff} \cdot OAC_{ff} \left( {950\;{\text{nm}}} \right),$$

where the constants $c_{wb}$ and $c_{ff}$ are related to the conversion factor (mass-specific absorption cross section (μg/m^2^)) between particulate mass concentration and optical absorption coefficient through an inverse relation. The conversion factors $c_{wb}$ and $c_{ff}$ were then estimated by linear regression of Eq. 6,^19,57^. However, accurate determination of $c_{wb}$ and $c_{ff}$ requires precise measurement of the CM, CM_ff_, and CM_wb_. Various methods are employed for these measurements. Some studies use BC data from Aethalometers at 880 nm to estimate the BC fraction of CM^16^. Others rely on thermoanalytical measurement techniques to measure organic carbon (OC), EC, and TC to estimate $c_{wb}$ and $c_{ff}$ values^48,49^. Additionally, accelerator mass spectrometry (AMS) or combinations of different instruments are used for this purpose as well^58^. The Aethalometer model has approximately 20% uncertainty when source-specific AAE values are verified with site-specific auxiliary measurements^55^. Given the complexities and poor time resolution measurements associated with AAE and CM determination, there is a pressing need for a simpler, real-time technique for source apportionment results.

#### The alternative spectral-based source apportionment

The proposed alternative spectral-based source apportionment method uses the same preliminary conditions, such as the dominant sources of carbonaceous emission being traffic and wood burning, as well as the AAE is the marker of the carbonaceous emission sources (Eqs. 2–5). In the first step of this approach, the source-specific AAEs are determined from the parallel measurements of size distribution and optical absorption^21^. Many earlier studies have experimentally demonstrated that under these measurement conditions, the size distribution of ambient aerosol can be fitted with two modes, where the particles fall into the modes with a count median diameter of about 30 nm (CMD_30_) originating dominantly from traffic activates, while the modes with CMD of about 130 nm (CMD_130_) include primary wood burning aerosols^21,59,60^. Based on this, the measured optical absorption at the ultimate, operational wavelengths of the Aethalometer is expressed by the following equations:

7 $$OAC\;(370\;{\text{nm}}) = N_{CMD30} \cdot SPOAC_{ff} \left( {370 \;{\text{nm}}} \right) + N_{CMD130 } \cdot SPOAC_{wb} \left( {370 \;{\text{nm}}} \right),$$

8 $$OAC\;(950\;{\text{nm}}) = N_{CMD30} \cdot SPOAC_{ff} \left( {950\;{\text{ nm}}} \right) + N_{CMD130 } \cdot SPOAC_{wb} \left( {950\; {\text{nm}}} \right),$$

where the N_CMD30_ and the N_CMD130_ are the number concentrations of traffic and wood burning aerosols of corresponding CMDs, while the SPOAC_ff_ and the SPOAC_wb_ are the hypothetical values of single particle OAC. Dividing Eqs. 7 and 8. yields the following equation:

9 $$\frac{{OAC\left( {370 \;{\text{nm}}} \right)}}{{OAC\left( {950\;{\text{ nm}}} \right)}} = \frac{{SPOAC_{ff} \left( {370\;{\text{ nm}}} \right) + \frac{{N_{CMD130} }}{{N_{CMD30} }} \cdot SPOAC_{wb} \left( {370 \;{\text{nm}}} \right)}}{{SPOAC_{ff} \left( {950\;{\text{nm}}} \right) + \frac{{N_{CMD130} }}{{N_{CMD30} }} \cdot SPOAC_{wb} \left( {950 \;{\text{nm}}} \right)}}$$

Depicting the ratio of OAC(370 nm)/OAC(950 nm) in the function of N_CMD130_/N_CMD30_ and fitting the measured data in the form of Eq. 9, the source-specific AAE values can be determined as the ultimate limits of the fitting curve (Eqs. 10 and 11).

10 $$\mathop {\lim }\limits_{{\frac{{N_{CMD130nm} }}{{N_{CMD30nm} }} \to 0}} \frac{{OAC\left( {370\;{\text{nm}}} \right)}}{{OAC\left( {950\;{\text{nm}}} \right)}} = \frac{{SPOAC_{ff} \left( {370\;{\text{nm}}} \right)}}{{SPOAC_{ff} \left( {950\;{\text{nm}}} \right)}} = \left( {\frac{370}{{950}}} \right)^{{ - AAE_{ff} }}$$

11 $$\mathop {\lim }\limits_{{\frac{{N_{CMD130nm} }}{{N_{CMD30nm} }} \to \infty }} \frac{{OAC\left( {370\;{\text{nm}}} \right)}}{{OAC\left( {950\;{\text{nm}}} \right)}} = \frac{{SPOAC_{wb} \left( {370\;{\text{nm}}} \right)}}{{SPOAC_{wb} \left( {950\;{\text{nm}}} \right)}} = \left( {\frac{370}{{950}}} \right)^{{ - AAE_{wb} }}$$

Employing the AAE_ff_ and the AAE_wb_ data, the contribution of fossil fuel and wood-burning aerosol absorption to the measured one at a given wavelength can be directly determined from Eqs. 2–5. The next step of this evaluation protocol is the determination of CM_ff_ and CM_wb_. Under these measurement conditions, the measured OAC and the source-specific AAE as a function of mass-specific absorption coefficient can be expressed as:

12 $$OAC\left( {370\;{\text{ nm}}} \right) = CM_{ff} \cdot \sigma_{ff} \left( {370{\text{ nm}}} \right) + CM_{wb} \cdot \sigma_{wb} \left( {370{\text{ nm}}} \right)$$

13 $$OAC\left( {950\;{\text{ nm}}} \right) = CM_{ff} \cdot \sigma_{ff} \left( {950\;{\text{ nm}}} \right) + CM_{wb} \cdot \sigma_{wb} \left( {950\;{\text{ nm}}} \right)$$

14 $$\frac{{\sigma _{{ff}} \left( {370{\text{ nm}}} \right)}}{{\sigma _{{ff}} \left( {950{\text{ nm}}} \right)}} = \left( {\frac{{370}}{{950}}} \right)^{{ - AAE_{{ff}} }}$$

15 $$\frac{{\sigma _{{wb}} \left( {370{\text{ nm}}} \right)}}{{\sigma _{{wb}} \left( {950{\text{ nm}}} \right)}} = \left( {\frac{{370}}{{950}}} \right)^{{ - AAE_{{wb}} }}$$

The AAE for aerosols originating from fossil fuel combustion and wood burning can be derived by leveraging the correlation between the optical absorption coefficient and the measured aerosol size distribution^21^. This relationship allows the system of equations (Eqs. 2–5) to be solved analytically, as the four equations now correspond to four unknowns. Given ${\text{OAC}}_{{{\text{ff}}}} \left( {950{\text{ nm}}} \right)$ obtained from solving Eqs. 2–5, along with the known AAE_ff_ and the mass-specific absorption cross section of fossil fuel aerosol at an arbitrary wavelength ($\sigma_{{{\text{ff}}}} \left( {\lambda_{0} } \right)$), the CM_ff_ can be defined as:

16 $$CM_{{ff}} = {\text{ }}\frac{{OAC_{{ff}} \left( {950\;{\text{nm}}} \right)}}{{\sigma _{{ff}} \left( {\lambda _{0} } \right)}}\left( {\frac{{\lambda _{0} }}{{950}}} \right)^{{ - AAE_{{ff}} }}$$

Similarly, the CM_wb_ can be expressed from the rearrangement of Eq. 12:

17 $$CM_{wb} = \frac{{OAC\left( {370\;{\text{ nm}}} \right) - CM_{ff} \sigma_{ff} \left( {370\;{\text{ nm}}} \right)}}{{ \sigma_{wb} \left( {370 \;{\text{nm}}} \right)}}$$

To determine CM_wb_, we use the thumb-of-rule relation between the CM and EC. Many earlier studies have demonstrated experimentally that the EC_ff_ content of CM_ff_ is about 50%, while the CM_wb_ comprises around 1/3 EC_wb_ content^61–63^. Using these relations, the EC_wb_ and the CM_wb_ can be expressed as:

18 $$EC_{wb} = EC - EC_{ff} = EC - \frac{1}{2}CM_{ff}$$

19 $$CM_{wb} = 3\left( {EC - \frac{1}{2}CM_{ff} } \right)$$

Therefore, using the synergy of thumb-of-rule-relation between the CM and EC content of the emission sources and the measured size distribution and optical absorption coefficient data, the proposed model provides a straightforward opportunity for real-time and selective source apportionment of the emission sources of carbonaceous particulate matter of the atmosphere.

## Results

### Diurnal variation and wavelength dependency of AAE

**Fig. 1:**
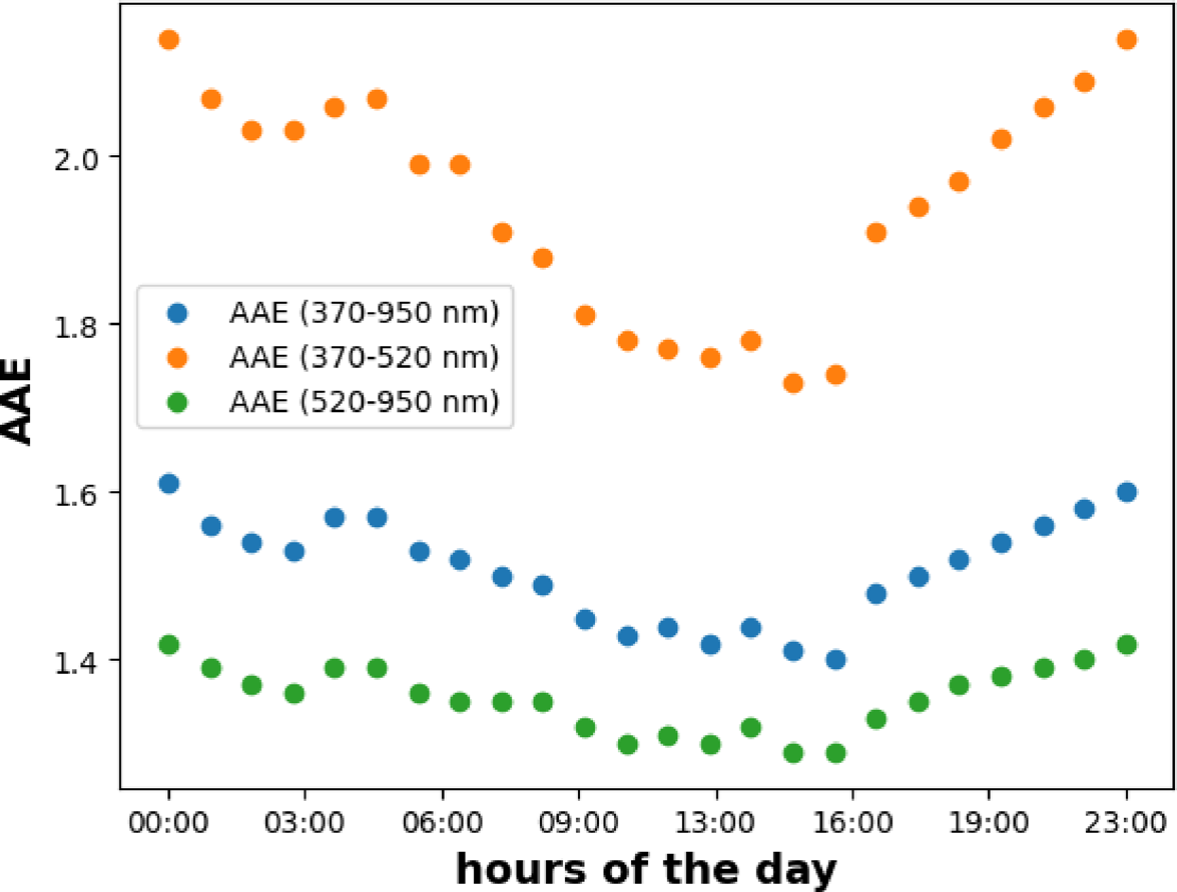
The diurnal variation of AAE at the specified wavelength pair of the Aethalometer.

The daily variation of the AAE inferred from optical absorption measurements obtained by the Aethalometer is illustrated in Fig. 1. A generally used wavelength-independent multiple scattering correction factor of 2.14 was employed in the calculation of OAC^25^. The AAE data were averaged for each hour of the day for 2 months. We have identified two distinct dynamics of the AAEs corresponding to daily variations in the carbonaceous mass of aerosols, observed in the wavelength ranges of 370–520 nm and 520–950 nm. Additionally, a third wavelength-independent AAE approximation (370–950 nm), widely used, is also depicted in Fig. 1. Inside these domains the AAE is independent of the applied wavelength pairs and shows similar trends but with different AAE values and dynamics^64^ (Fig. 1). Although the trends of AAE is similar, higher dynamics AAE have been observed at the lower wavelength domain pair than that of the higher wavelength pair (Fig. 1). This phenomenon arises from the characteristics of organic matter present in wood burning aerosols, which demonstrate minimal absorption within the visible to near-infrared wavelength domain resulting in lower AAE values. Conversely, it exhibits the highest absorption within the ultraviolet spectrum domain, leading to higher AAE values in the UV–visible range^21,22^ (Fig. 1).

**Fig. 2:**
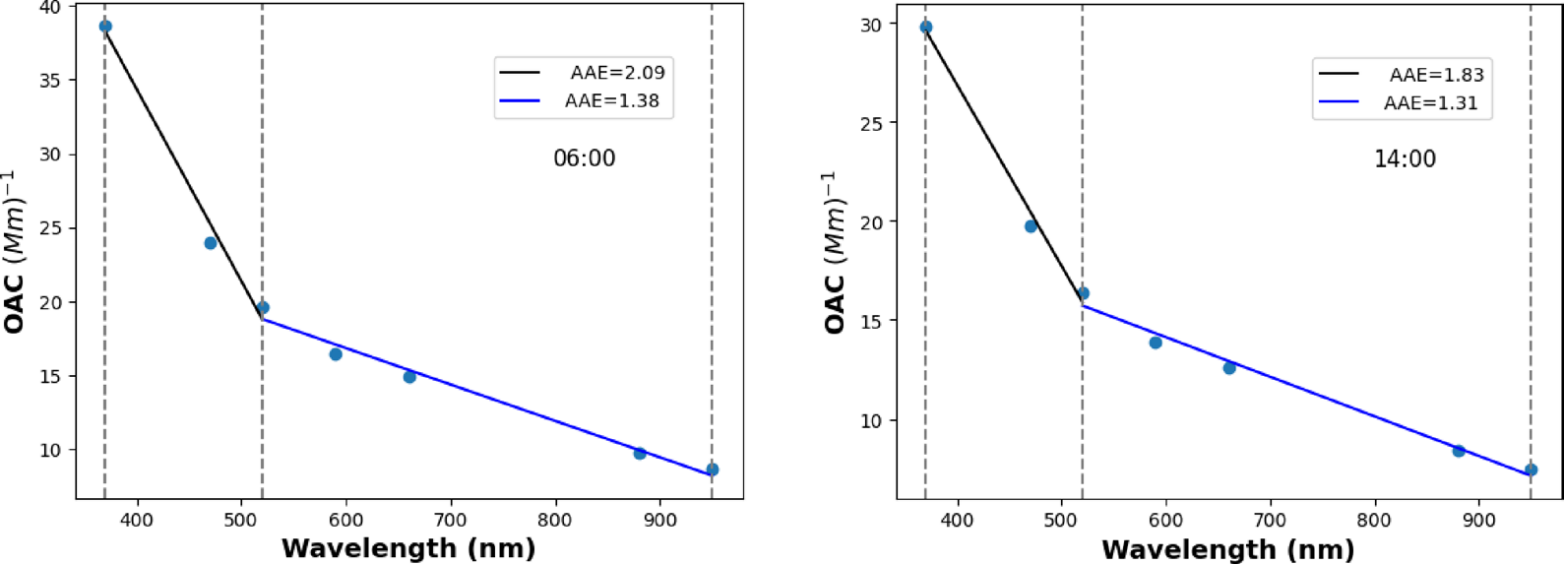
The wavelength-dependent optical absorption quantified by AAE at two representative periods of the day (06:00 and 14:00).

This type of daily dynamics of AAE agrees well with many earlier studies’ results^20,59^. These dynamics of AAE align closely with the distinctive properties of aerosols originating from their respective sources. The aerosols dominated by traffic activities have the lowest AAE, i.e., nearly 1, while the aerosols dominated by wood burning have higher AAE values^5,7,65^. The daily dynamics of the AAE are primarily driven by the emission strengths of the sources^61^. In the daytime, when traffic activities are dominant, the AAE is lower, while in the late afternoon and at night, when residential heating is prevailing, the AAE is increased. The steep drop in AAE values around 15:00–17:00 shows the abundance of vehicles on the road. The measured optical absorption and the AAE values are depicted in the two representative periods of the day (Fig. 2). At 6:00 AM, when the residential heating activities are dominant, the AAE value is higher than 2, while at 14:00 when the relative contribution of traffic aerosol is increased, it decreased to around 1.8 in the 370–520 nm wavelength domain. However, in the 520–950 nm wavelength range, the AAE shows negligible deviations in these two periods of the day. Although many earlier studies using free-floating (filter-free) samples have experimentally demonstrated the wavelength dependency of AAE, this study provides the first experimental evidence of this phenomenon using optical absorption measurement based on the transmission measurement of the filter-accumulated aerosol (Fig. 2).

### The measured size distribution and its mode structure

**Fig. 3:**
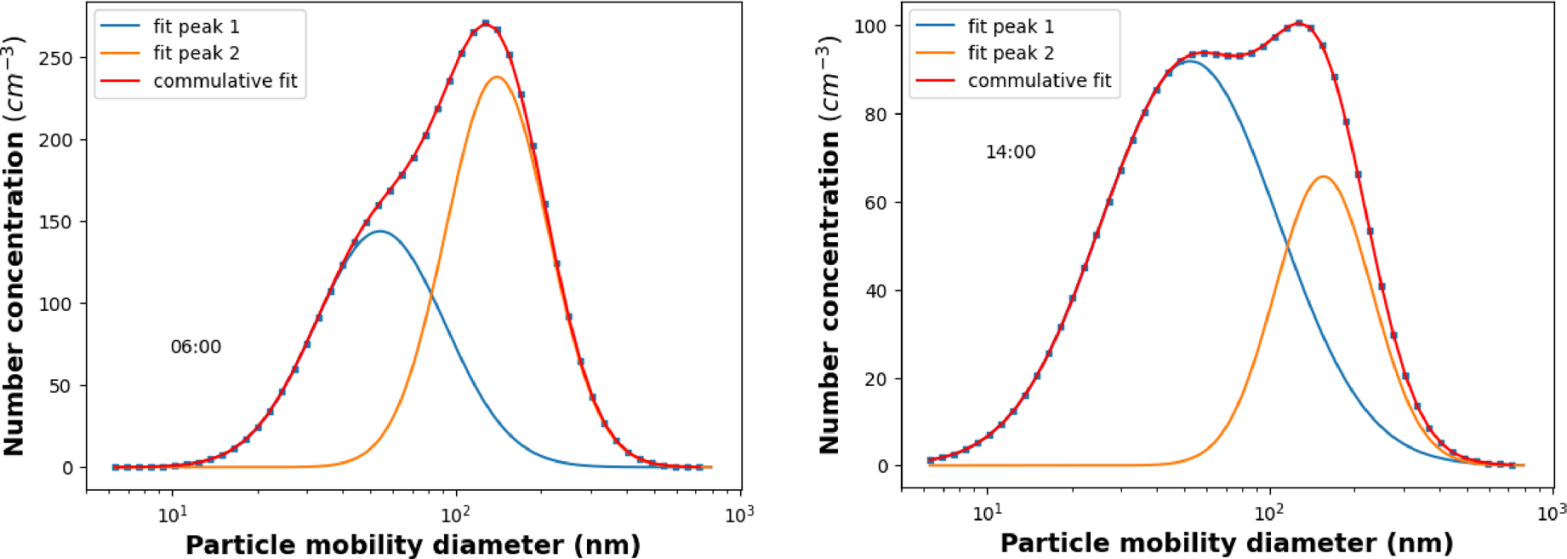
The measured size distribution followed by mode structure for two representative periods of the day (Fig. 1).

The particle size is the key parameter in the transport and deposition of ambient aerosols. Figure 3 presents the number–size distributions of aerosol particles using data from DMPS. We applied the lognormal multipeak fitting method on measured size distribution data to determine the bi-modal size distribution of the ambient aerosols^66–72^. The data is averaged over each hour of the day. Each multi-peak lognormal fitting revealed a bi-modal structure, with modal sizes falling within the ranges of 20–40 nm and 90–170 nm for the first and second modes. The aerosol falls into the modes having the count median diameter (CMD) between 20 and 40 nm is referred to as CMD_30_, while the mode dominated by larger size particles with higher CMD values around 130 nm is termed as CMD_130_. Furthermore, the convolution of the two identified modes gives the measured size distribution back with high accuracy, which further confirms the reliability of the assumption that the two sources can be segregated from each other through the bimodal fitting procedure. The bi-modal size distributions for the two representative periods of the day are shown in Fig. 3. Based on the earlier literature studies, it is experimentally verified that the aerosol that falls into the CMD_30_ modes dominantly originated from traffic emission, while in the CMD_130_ mode, the wood burning aerosol is decisive in rural areas^66,67,69,70^. One can see from Fig. 3. that the emission strengths represented by the ratio of the total number concentration of the modes (TNC) of the two sources differ significantly from each other in the depicted representative periods of the day (Fig. 3). At 6:00, the aerosol number concentration falls into the CMD_130_ mode is higher than that of CMD_30_ mode. On the contrary, during the afternoon, the number concentration of the CMD_30_ becomes more prominent, indicating the relative increase of the traffic-related aerosol emissions.

### The correlation between measured absorption and thermoanalytical data

**Fig. 4:**
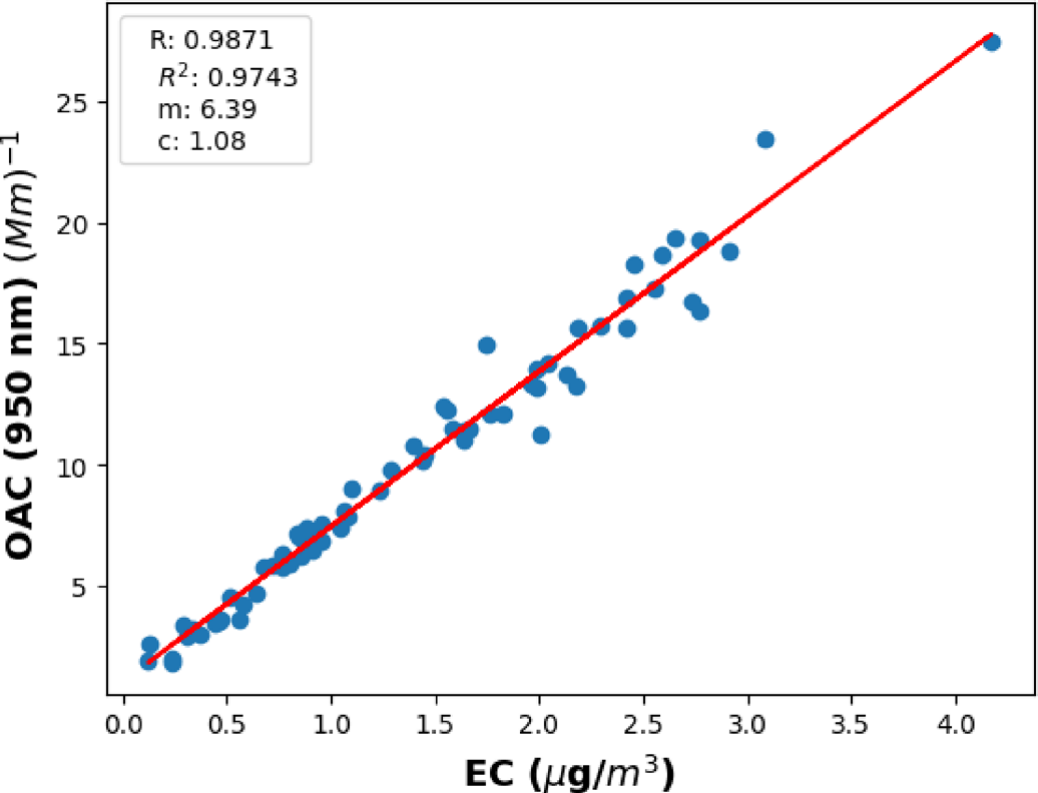
The optical absorption calculated at highest wavelength of Aethalometer versus elementary carbon measurements from OC/EC analyzer.

We obtained the elemental carbon data from OC/EC analyzer with 6-h average and the measured data is plotted as a function of optical absorption at the longer operational wavelength of the Aethalometer (950 nm) (Fig. 4). The OAC data is averaged and synchronized with the same sampling period for this correlation. The correlation between measured OAC and EC values offers opportunities to calculate the mass-specific absorption cross-sections of the EC aerosols originating from traffic and wood burning emissions. The identified linear correlation is quantified by its slope, the coefficient of correlation (R), and the coefficient of determination (R^2^) (Fig. 4). The slope of the plot gives the mass-specific absorption cross section of EC content of the measured aerosol assembly at the specified wavelength of the Aethalometer, $\sigma_{EC} \left( {950 \;{\text{nm}}} \right) = 6.39$ (m^2^/µg). The strong correlation of 0.99 between the measured quantities highlights the reliability of this relationship in the determination of $\sigma_{EC}$.

**Fig. 5:**
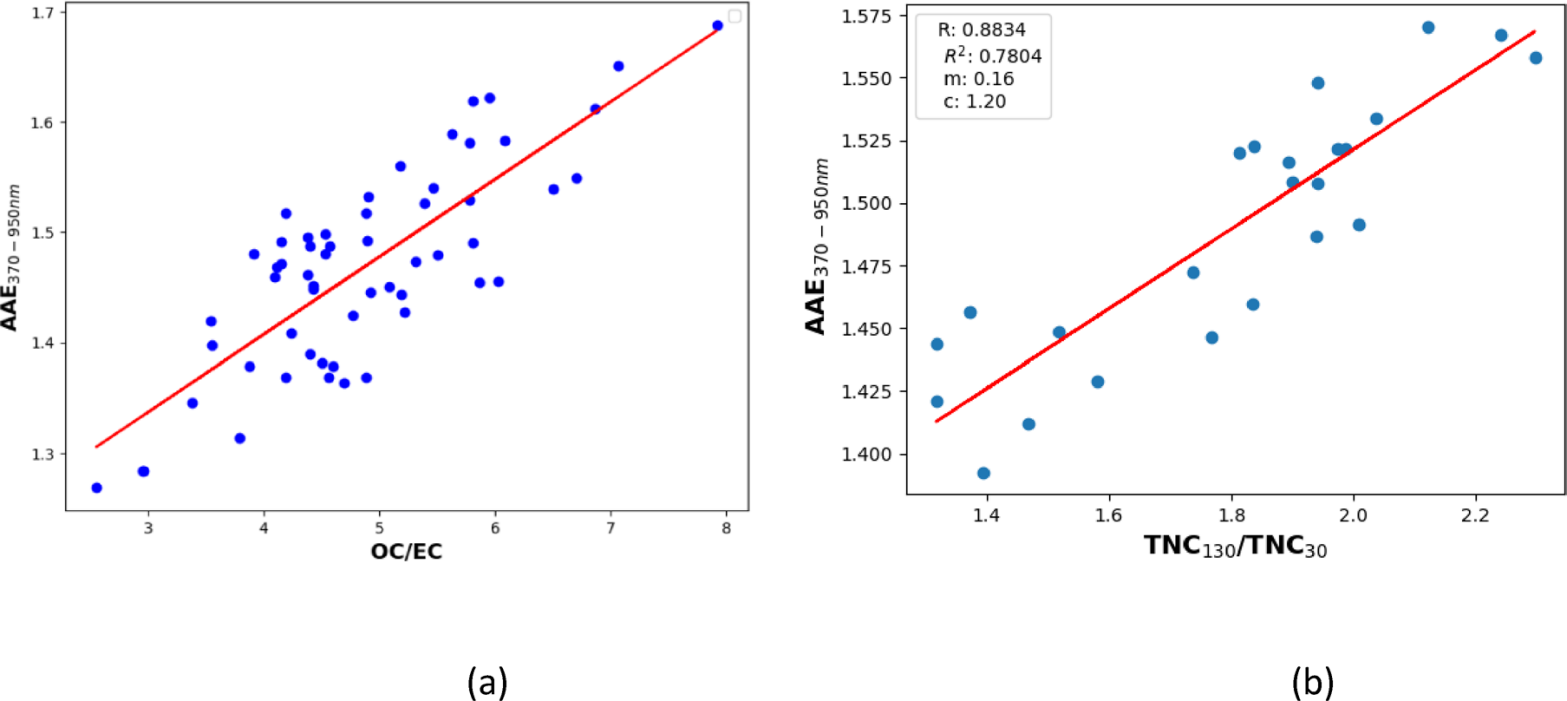
The AAE obtained and its corelation as function of (**a**) OC/EC measurements (**b**) mode-structure.

The correlation and quantification of AAE with a ratio of measured size modes and OC/EC have also been demonstrated (Fig. 5a–b). The OC/EC data is directly obtained from OC/EC analyzer, and its correlation with the AAE emphasizes the significant influence of aerosol chemical composition on AAE values^59,73^. The OC fraction is more dominant in wood burning than in fossil fuel aerosol, therefore, the OC/EC ratio carries information regarding the relative strengths of wood burning in the measured aerosol assembly^59^. Moreover, the identified correlation between the AAE and the OC/EC ratio opens novel possibilities to measure the relative strengths of OC/EC in real time through the measured optical absorption data^73^. The slope of OC/EC correlation is 0.07, and the correlation coefficient and the standard deviation of the fitting were found to be 0.8 and 0.6, respectively. The strong correlation of the ratio of the modes TNC_130_/TNC_30_ with AAE implies that both the diurnal dynamics of AAE and mode structure are driven by the emission strength of different sources associated with traffic and heating activities.

### Source apportionment with Aethalometer and the proposed model

**Fig. 6:**
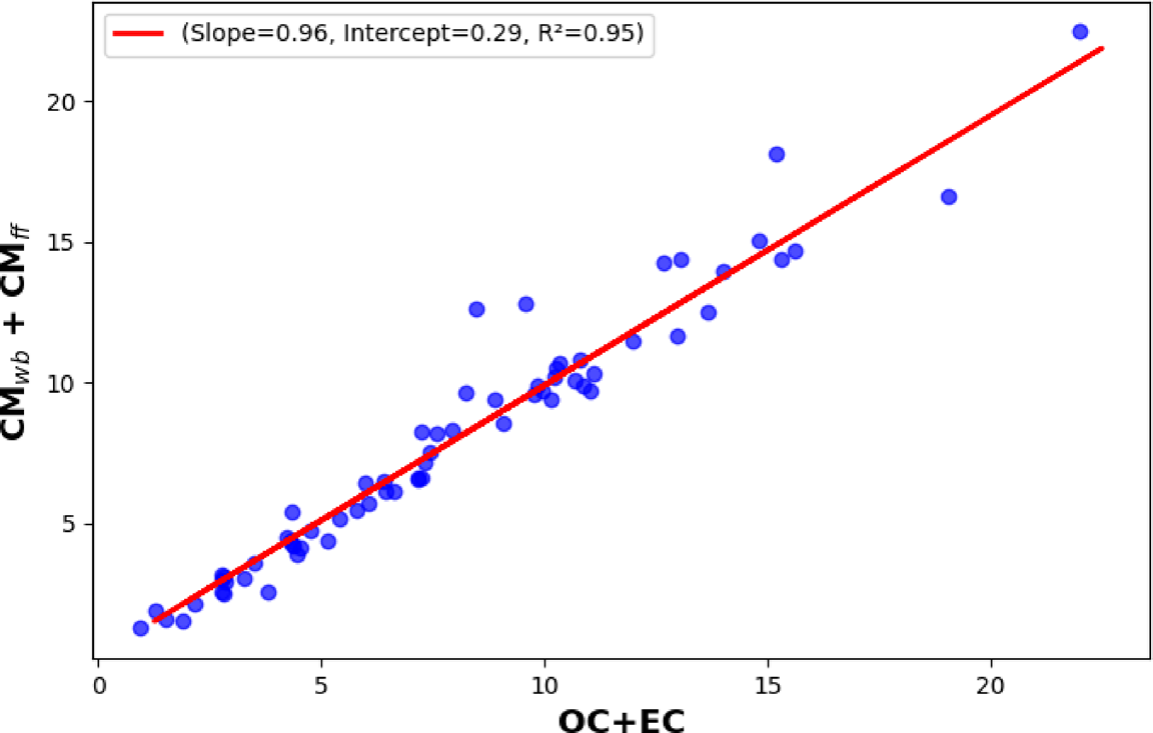
Scatter plot of OC + EC versus estimated CM_wb_ + CM_ff_ showing the slop of 0.96 with R^2^ = 0.95.

First, the source apportionment results were obtained using the originally proposed Sandradewi model or the so-called Aethalometer model^19^. The total carbonaceous particulate matter was obtained simply by using the OC/EC analyser measurement data here. The AAE_ff_ was set fixed at 1.1 while AAE_wb_ was varied between 1.1 and the maximum value of the measured AAE data. Then for each AAE_wb_ value the linear regression was performed to obtain conversion coefficients c_ff_ and c_wb_, thereby estimating the mass-specific optical absorption of two aerosol types in Eq. 6. The c_ff_ and c_wb_ values were then used to calculate CM_wb_ and CM_ff_ using Eq. 6. The optimal AAE_wb_ value was selected based on the best 1:1 correlation between the estimated CM_wb_ + CM_ff_ and the thermoanalytically measured OC + EC data (Fig. 6). Based on this calibration procedure the AAE_wb_ is found 1.69 ± 0.2 which is well agreed with the previously reported values^73,74^. From the scatterplot of CM_wb_ + CM_ff_ in the function of OC + EC data, we obtain a slope of 0.96 ± 0.1 with a correlation coefficient of 0.95, which further confirms the reliability of the applied model.

**Fig. 7:**
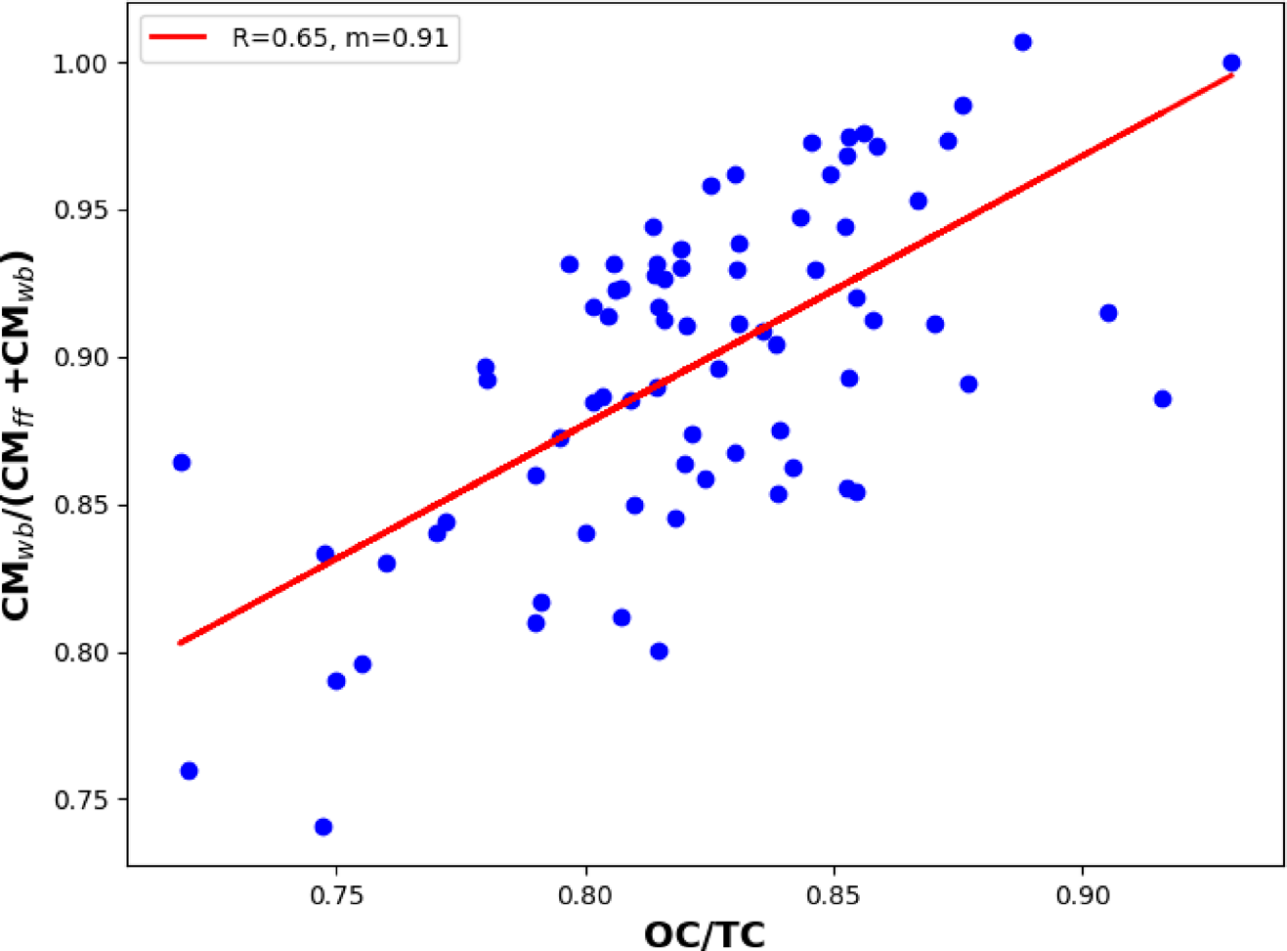
The scatter plot of the CM_wb_/CM_tot_ versus OC/TC data obtained from OCEC analyzer.

Since the OC fraction of the emitted carbonaceous particulates is more dominant in wood burning than in traffic aerosol, the relative strengths of the wood burning emission can be represented by the estimated CM_wb_/(CM_wb_ + CM_ff_) ratio^19^. The scatterplot of the CM_wb_/(CM_wb_ + CM_ff_) in the function of OC/TC ratio has shown a good correlation between the measured quantities using AAE_ff_ = 1.1 and AAE_wb_ = 1.69 in the data evaluation procedure (Fig. 7). The slope of the fitted curve is 0.91 ± 0.1 with the correlation coefficient of 0.65 which yields a determination coefficient of 0.43.

**Fig. 8:**
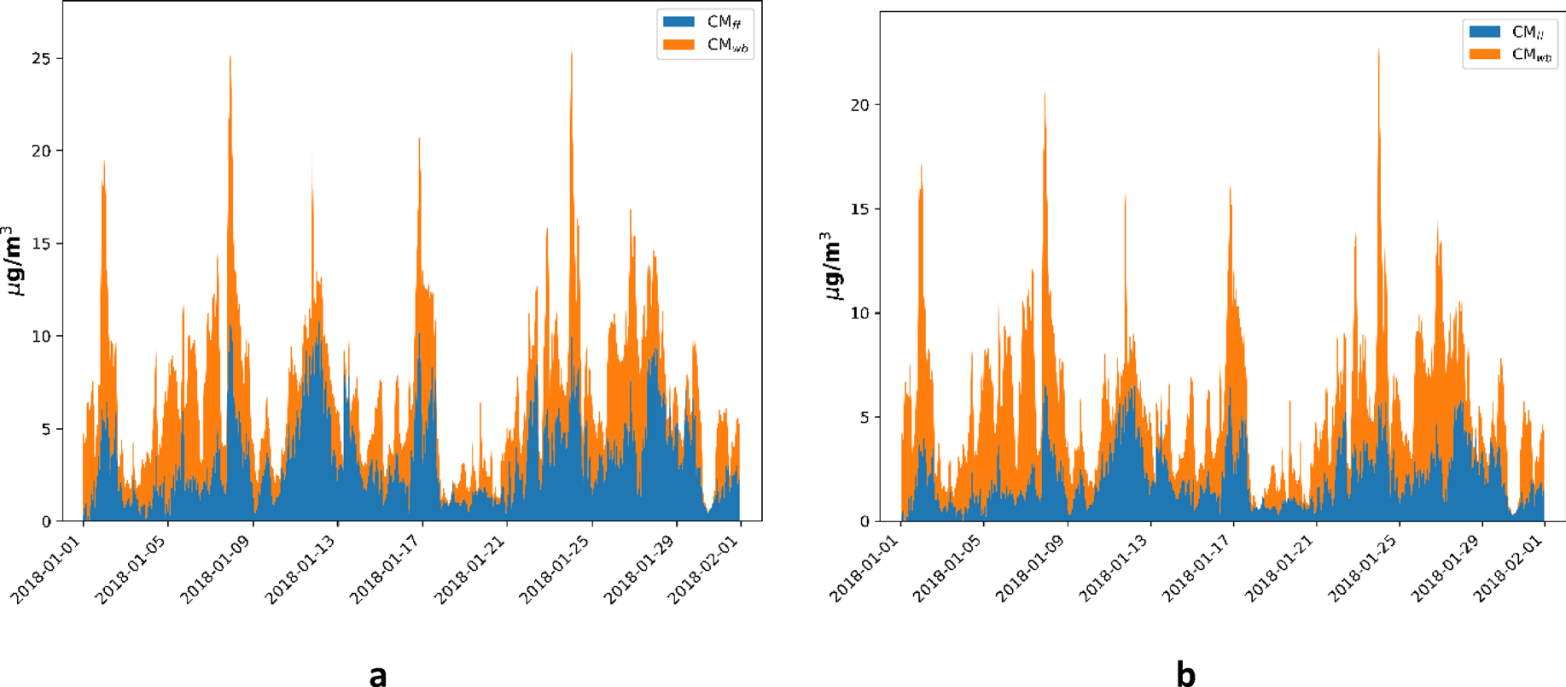
The time series of mass concentrations of fossil fuel and wood burning aerosols estimated from the Aethalometer measured data using different assumptions (**a**) using site specific, experimentally determined AAE and wavelength-independent multiple scattering correction factor (C = 2.14) (**b**)employing commonly used AAE values (AAE_ff_ = 1 and AAE_wb_ = 2) alongside a wavelength-independent multiple scattering correction factor (C = 2.14).

The mass concentration of the fossil fuel and wood-burning aerosols using site-specific AAE values along with a wavelength-independent multiple scattering correction factor is shown in Fig. 8a. For comparison purposes, the source apportionment results using generally accepted values of AAE_ff_ = 1 and AAE_wb_ = 2 are also presented in Fig. 8b. The wavelength-independent multiple scattering correction factor of 2.14 was used for the calculations^25^ (Fig. 8).

In the proposed, alternative source apportionment model, the determination of aerosol mass concentrations originating from traffic and wood-burning emission is deduced from the site and source-specific AAE values defined by the correlation between the mode structure and the measured optical absorption coefficient (described in Sect. 3.3.) and the thumb-of-rule relation between EC and TC of the traffic and wood burning emission (Eqs. 18–19).

**Fig. 9:**
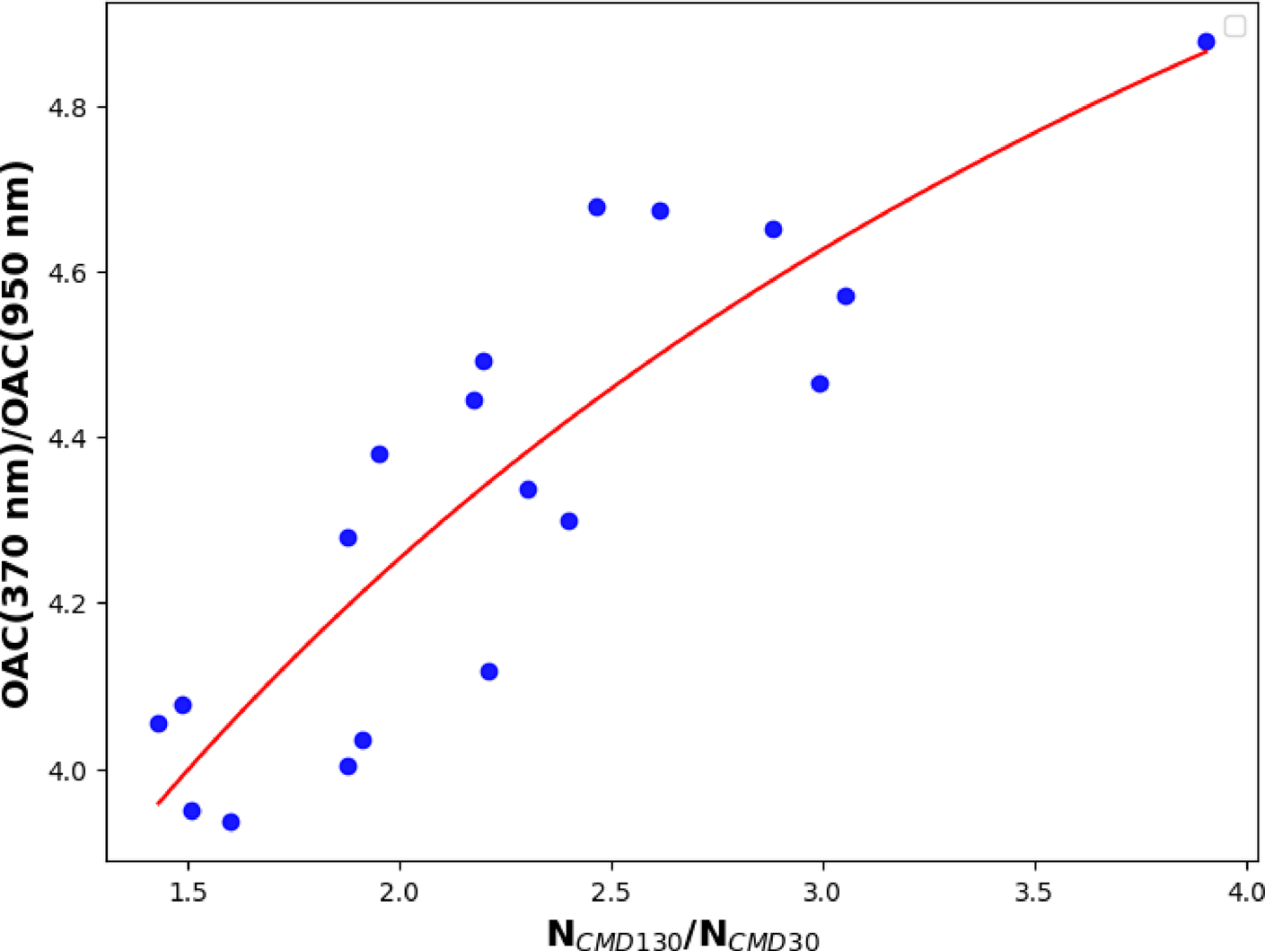
The ratio of the measured optical absorption coefficient in the function of the ratio of the number concentration of the two identified modes.

Based on the experimentally verified assumption that when the traffic and wood-burning aerosols are the dominant emission sources of carbonaceous emission, the two sources can be identified through their bimodal size distribution deduced from the measured size distribution data of ambient aerosols (Fig. 3). Take advantage of parallel measurements of size distribution and optical absorption, the source-specific AAEs can be defined through Eqs. 9–11. The correlation between the ratio of the optical absorption coefficient measured at the ultimate wavelengths of the instrument in the function of the ratio of the number concentration falls into the two identified modes, is depicted in Fig. 9.

Defining the fitting line based on the form of Eq. 9, the source-specific AAE values can be determined as the ultimate limits of the fitted curve shown in Fig. 9. In this limit, when the N_CMD130_/N_CMD30_ converges to 0 means that the measured light absorption dominantly corresponds to the traffic activities, while when the N_CMD130_/N_CMD30_ converges to infinity means that the contribution of wood burning aerosol to the light absorption is dominant (Eqs. 10 and 11). Based on this extrapolation, the AAE_ff_ and AAE_wb_ are 1.03 ± 0.1.and 1.96 ± 0.2, respectively. These AAE values agree well with the general exception and the earlier experimental results^16^. The correlation coefficient and the standard deviation of the fitting were found to be 0.87 and 0.15, respectively.

**Fig. 10:**
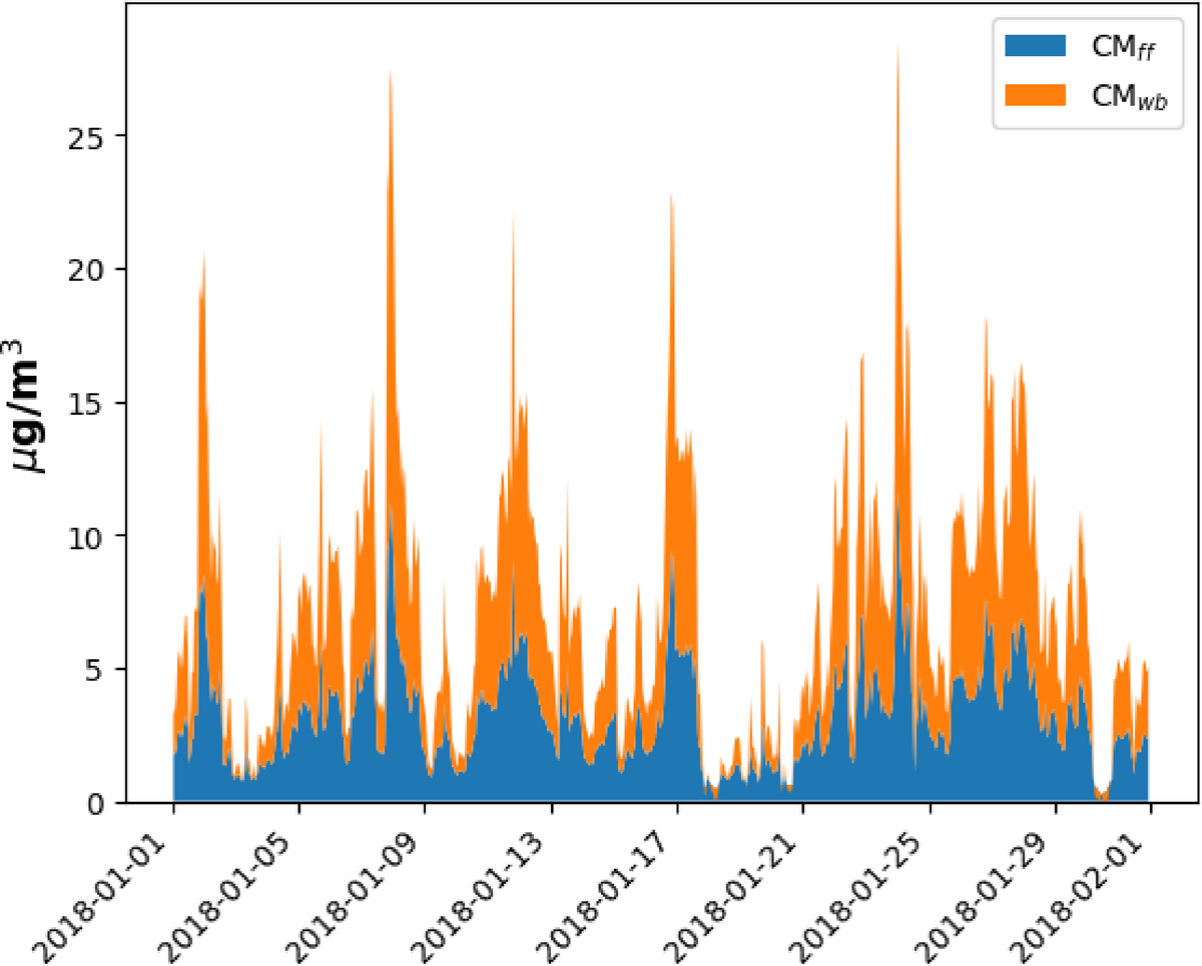
The time series of mass concentrations of fossil fuel and wood burning aerosols estimated from the improved proposed model.

Using the defined AAE values the contribution of the wood burning and traffic aerosol to the measured one at any specific wavelength can be determined from Eqs. 2–5. Using the AAE_ff_ value of 1.03, the CM_ff_ can be calculated from Eq. 10. In this calculation the default value of mass-specific absorption cross section i.e., $\sigma_{{{\text{ff}}}} \left( {550\;{\text{nm}}} \right) = 7.5{\text{ g/m}}^{2} { }$ was used^75–77^. The CM_wb_ was calculated by using thermoanalytically measured EC data and the thumb-of-the-rule relation between the EC and TC ratio of the two sources (Eq. 11). Based on this approach, the source apportionment results obtained from the proposed model are depicted in Fig. 10.

## Summary and discussion

The parallel measurement of size distribution and absorption response of the ambient was measured in this study. Although the wavelength dependency of the measured AAE was experimentally demonstrated in many earlier studies^59,78^ using filter accumulated transmission measurement for the determination of the optical absorption coefficient of the ambient aerosols, we demonstrated here for the first time that the measured OAC from filter sampling can be characterized by two AAEs. One is related to the 390–550 nm, while the other is associated with 550–920 nm wavelength domain. The absolute value of the AAE and its dynamics increase towards the shorter wavelength range (Figs. 1 and 2). We also further experimentally confirmed that the size distribution of the measured aerosol assembly can be fitted with two log-normal unimodal modes, and each mode is characteristic of the emission sources of the carbonaceous particulate (Fig. 3). We also demonstrated the correlation between the real-time measured AAE and the off-line measured OC/EC ratio (Fig. 5b). Using the site and source-specific slope of this correlation as a calibration factor, this correlation makes it possible to measure the OC/EC ratio in real time through the measurement of the wavelength dependency of the absorption. Since the AAE depends on the applied wavelength pair, the sensitivity of the AAE for the measurement of the OC/EC ratio is changed when the AAE is calculated using a different wavelength domain (Table 1).

**Table 1:** The slope, the standard deviation, and the correlation coefficient deduced from the correlation between the AAE and the OC/EC ratio.

AAE (Wavelength-pair)	OC/EC (Slope)	OC/EC (Standard deviation)	OC/EC (Corelation coefficient)
370–950	0.07	0.0546	0.8
370–520	0.06	0.045	0.88
520–950	0.3	0.65	0.65

One can realize from Table 1, that the stronger correlation of evaluated AAE with measured OC/EC increased towards the shorter wavelength domain (higher correlation coefficient and lower standard deviation). The highest correlation can be found in the 370–520 nm wavelength range.

The Aethalometer and the proposed source apportionment model were used to determine the CM_ff_ and CM_wb_ of ambient in real-time (“Source apportionment with Aethalometer and the proposed model” Section). Using the Aethalometer model, the thermoanalytically measured TC data were applied to determine the source-specific AAE of the different sources^19^. Alternatively, the commonly used AAE_ff_ = 1 and AAE_wb_ = 2 was also used to determine the CM_ff_ and CM_wb_ in the Aethalometer model^52,53^. Although the results of the two approaches show similar dynamics, the average and the ratio of the different sources calculated for the whole measurement period differ from each other (Table 2).

**Table 2:** The average and the ratio of PM_ff_ and PM_wb_ concentrations using different approaches.

	AAE_ff_	AAE_wb_	CM_ff_ (μg/m^3^) (avg)	CM_wb_ (μg/m^3^) (avg)
Aethalometer-model using site-specific AAE	1.1	1.69	2.87	4.1
Aethalometer-model using default AAE values	1	2	2.33	3.36
Proposed approach using site-specific AAE	1.03	1.96	2.99	3.98

This discrepancy underscores the importance of incorporating site-specific characteristics for source apportionment studies. The necessity for a straightforward, on-site measurement technique for source apportionment results arises from minor fluctuations in AAE leads to substantial changes in source apportionment results. Such a technique should ensure efficient and reliable source apportionment results while minimizing time requirements and simplifying data evaluation. Hence, the demonstration of our improved model is important to address this pressing need.

In the proposed spectral-based source apportionment model, the source-specific AAE data is determined by the correlation between the ratio of the total number concentration of the aerosol falls into the different modes and the ratio of the optical absorption measured at the ultimate operational wavelengths of the Aethalometer^21^ (Fig. 9). Using this approach, the AAE_ff_ and the AAE_wb_ are 1.03 ± 0.2 and 1.96 ± 0.15 respectively. Applying the AAE data and the proposed thumb-of-rule relation between the EC and TC of the different sources, we have calculated the PM_ff_ and the PM_wb_ (Fig. 10). The correspondence in mass-specific absorption cross-section values between the improved model and the originally defined Aethalometer model underscores the validity of our proposed model. The percentage difference between outcomes of Aethalometer-model by employing site-specific AAE values and by employing the simplified assumptions of AAE results in 21% and 20% for traffic and wood burning aerosols respectively (Table 2). The outputs of the classical Aethalometer model using site-specific AAE values and the proposed model are closely related (Table 2). The percentage difference between the outputs of the two models is 4% and 3% for fossil fuel and wood-burning aerosols respectively.

In this study, we demonstrate a simplified alternative source apportionment model utilizing real-time, parallel measurements of size distribution and optical absorption data using DMPS and Aethalometer. The model is based on an empirical relationship between TC and EC for different sources. Additionally, we examined the wavelength dependence of the AAE derived from the measured OAC from the Aethalometer. The source apportionment results from our model were compared with those from the conventional Aethalometer model. The percentage difference between the proposed model output and the originally introduced Aethalometer model output is less than 10% at each measurement point, which further validates the proposed alternative model.

## Data Availability

The datasets used and/or analysed during the current study available from the corresponding author on reasonable request.
